# Colibri® insecticide induces male reproductive toxicity: alleviating effects of *Lannea acida* (Anacardiaceae) in rats

**DOI:** 10.1186/s12610-019-0096-4

**Published:** 2019-12-20

**Authors:** Aimé Césaire Momo Tetsatsi, Pepin Alango Nkeng-Effouet, Désiré Munyali Alumeti, Georges Roméo Fozin Bonsou, Albert Kamanyi, Pierre Watcho

**Affiliations:** 1 Animal Physiology and Phytopharmacology Laboratory, Faculty of Science, University of Dschang, PO Box: 67, Dschang, Cameroon; 2 Department of Chemistry, Faculty of Science, University of Dschang, Dschang, Cameroon

**Keywords:** Colibri®, Reproductive toxicity, Oxidative stress, *Lannea acida*, Rat, Colibri®, Toxicité reproducitive, Stress oxydatif, *Lannea acida*, Rat

## Abstract

**Background:**

Imidacloprid, a neonicotinoid insecticide, has been associated to severe reproductive toxicity in mammals. Although some preventive measures have been reported, curative strategies are yet to be explored. The present study was designed to investigate the alleviating effects of *Lannea acida* on the reproductive toxicity of colibri®, a commercial formulation of imidacloprid, in adult male rats.

**Materials and methods:**

Seventy rats were orally administered with colibri® (22.5 mg/kg, 10 mL/kg) for 14 days and treated for other 14 or 28 days with either aqueous or methanol extracts of *L. acida* (170 or 340 mg/kg). Control animals were similarly treated with clomiphene citrate or vitamin E. Sexual organ weights, spermatozoa characteristics, sexual hormones, stress markers and testis histology were evaluated at the end of each treatment period.

**Results:**

Colibri® exposition induced reproductive toxicity marked by a decrease in sex organ weights, spermatozoa count, motility and viability. Colibri® also decreased testosterone, luteinizing hormone, follicle stimulating hormone concentrations and increased testicular oxidative stress. Spermatozoa morphology and testis histology were also severely altered. Similar to clomiphene citrate and vitamin E, treatment with *L. acida* extracts significantly (*p* ≤ 0.05–0.001) reversed the above-mentioned damages, especially after 28 days of treatment with aqueous (340 mg/kg) and methanol (170 mg/kg) extracts.

**Conclusion:**

Present results indicate that *L. acida* exerts curative effects against colibri®-induced male reproductive toxicity. These results justify the use of this plant as fertility enhancer and suggest that it could be an alternative in the management of pesticide-derived male infertility.

## Résumé

**Contexte et justificatif:**

Les effets toxiques de l’imidaclopride; insecticide de la famille des néonicotinoïdes, sur la fertilité sont largement documentés. Cependant, les mesures curatives restent encore à explorer. L’objectif de ce travail était d’évaluer les effets curatifs de *Lannea acida* sur la toxicité reproductive du colibri®, une formulation commerciale de l’imidaclopride, chez le rat mâle.

**Matériel et méthodes:**

Soixante-dix rats ayant reçu par gavage le colibri® (22,5 mg/kg, 10 mL/kg) pendant 14 jours ont été répartis en groupes et traités pendant 14 ou 28 jours à l’extrait aqueux ou méthanolique de *L. acida* (170 ou 340 mg/kg)*.* Les groupes témoins recevaient le citrate de clomifène ou la vitamine E. Au terme de chaque période de traitement, quelques paramètres de fertilité ainsi que les marqueurs du stress et l’histologie testiculaire étaient évalués.

**Résultats:**

Une diminution significative de la masse des organes sexuels, de la densité, la motilité, la viabilité spermatiques et des hormones sexuelles (testostérone, LH et FSH) a été notée chez les animaux exposés au colibri®. Ce toxique a également généré le stress oxydatif et les dommages testiculaires. Tout comme le citrate de clomifène et la vitamine E, les extraits de plante ont significativement (*p* ≤ 0,05-0,001) réparé ces altérations, notamment aux doses de 340 et 170 mg/kg des extraits aqueux et méthanolique respectivement.

**Conclusion:**

Ces résultats renseignent que *L. acida* est. doué de propriétés curatives contre la toxicité reproductive du colibri®. Ceci suggère que cette plante serait une alternative prometteuse dans le traitement de l’infertilité masculine.

## Introduction

From 2.5 billion in 1950, the world population is estimated to reach 9.1 billion by 2050 [1]. This rapid growth is accompanied with the pressing challenge of food availability and security. For these reasons, the production and use of pesticides has spectacularly increased during recent decades. Pesticides are a broad group of chemicals that have benefits to human by increasing food productivity and decreasing food-borne and some vector-borne diseases [2]. However, pesticides have potent harmful effects to non-target organisms, including humans depending on the agent and the exposure [3]. Animals and humans are potentially exposed to pesticides either directly through occupational exposure or indirectly via food and water consumption [2]. Globally, about 5% of the population, mostly composed of agro-workers is directly exposed to insecticides including colibri® [4].

Colibri® is a broad-spectrum commercial insecticide used in many countries including Cameroon in pest control and seed treatment [5]. It is composed of only one active principle, imidacloprid which is the most important neuroactive insecticide in the market [6].

Imidacloprid (IMI) [1-(6-chloro-3-pyridylmethyl)-N-nitroimidazolidin-2-ylideneamine] is a chlorinated analogue of nicotine. The use of IMI is gradually increasing since its introduction into the market in 1992 due to its selective toxicity and apparent safety for humans [5]. Neonicotinoid insecticides act as agonists of insect nicotinic acetylcholine receptors (nAChRs) which play an important role in synaptic transmission in the central nervous system. Due to structural differences between mammal and insect nAChRs and the higher binding affinity for insect nAChR, IMI is considered a selective toxicant [7]. However, several studies have reported considerable damaging effects of this insecticide on rats nervous, excretory and reproductive systems. Indeed, a significant decrease in spermatozoa count, motility and viability associated with a decrease in sexual hormones and severe testis histological damages were reported in rats treated with IMI [5, 8, 9]. Oxidative stress which results from imbalance in the body’s oxidants and antioxidants in favor of the first is said to be the underlying mechanism generating these damages [10, 11].

As oxidative stress tends to be the common feature of many xenobiotic against human systems, extensive research activity has been performed during recent years to find effective and safety solutions, particularly natural compounds with antioxidant properties. In fact, the preventive effect of Broccoli and Curcumin against neurotoxicity and reproductive toxicity respectively of IMI has been reported in rat [12, 13]. However, less is known about the curative potentials of medicinal plants on the reproductive impairments due to IMI. *L. acida* (Anacardiaceae) is a medicinal plant traditionally used as fertility enhancer in many sub-Saharan countries including Burkina Faso, Nigeria and Cameroon [14]. Previous study approved the capacity of the methanol extract of this plant to improve sexual hormones, spermatozoa quality and quantity in normal rats [15]. Moreover, the antioxidant properties of the aqueous extract of *L. acida* were also reported in healthy male rats [16]. Considering the deleterious sexual effects of pesticides reported in farmers and inhabitants of farming areas [17], it was of great interest to find out whether these medicinal plant potentials could be helpful in reversing the male reprotoxicity due to colibri®. The present study was therefore undertaken to evaluate the alleviating effects of aqueous and methanol extracts of *L. acida* on spermatozoa deficiency, reproductive hormones and oxidative stress markers in colibri®-exposed rats.

## Materials and methods

### Chemical

Colibri® (Sun Valley Hall Limited-Hong Kong) is an insecticide containing IMI at 30 g/l as the only active principle. One liter of this compound (Bach N° SVH161104) was purchased from the Dschang market and, the working solution prepared in distilled water. Assay kits for testosterone, LH and FSH (Accubind, Monobind. Lake Forest, USA) were used according to the manufacturer’s instructions. All other chemicals and reagents were of analytical grade and purchased from local suppliers.

### Plant harvesting and authentication

The stem barks of *L. acida* were harvested in Malantouen, Noun Division, West Region of Cameroon. The plant sample was authenticated at the Cameroon National Herbarium, in comparison to the specimen deposited under the Voucher number 40942HNC. The plant material was shade-dried and grinded prior to aqueous and methanol extracts preparation.

### Aqueous and methanol extract’s preparation

The aqueous extract was prepared by decoction of 250 g of the powder in 1.5 L of boiled distilled water for 10 min. The solution was allowed to cool at room temperature and the filtrate was oven-dried to obtain 10.2 g of the aqueous extract. The extraction yield was calculated to be 4.08%. The methanol extract was prepared by maceration of 250 g of the powder of *L. acida* stem barks in 1 L of methanol for 72 h at room temperature. The filtrate was evaporated under reduced pressure and oven-dried to obtain 13.5 g of the methanol extract, giving an extraction yield of 5.4%. Each working plant extract solution was prepared in distilled water and the volume adjusted to 10 ml/kg.

### Animal care

Adult male Wistar rats weighing 150–200 g were obtained from the animal house of the Department of Animal Biology, Faculty of Science, University of Dschang, Cameroon. They were housed in plastic cages (4/cage) at room temperature with a natural light/dark cycle. Animals received standard rat diet and tap water ad libitum*.*

### Animal treatment

Seventy (70) adult male albino rats were daily given colibri® per os at the dose of 22.5 mg/kg IMI (10 mL/kg) daily during 14 days. Ten other rats constituted the normal untreated group and were orally given distilled water (10 mL/kg). After 14 days of continuous gavage, insecticide-treated rats were randomly partitioned into 7 groups of ten animals each and further treated for 14 or 28 days as follows: Group 1 served as negative control and was administered with distilled water (10 ml/kg), groups 2 and 3 were positive controls treated with clomiphene citrate (2 mg/kg per day) and vitamin E (75 mg/kg per day) respectively. Groups 4-5 and 6-7 constituted test groups and were respectively administered with aqueous or methanol extract of *L. acida* at the doses of 170 mg/kg or 340 mg/kg per day. All animals were daily weighed and the volume of each working solution was monitored accordingly.

The dose of IMI was selected from a screening test (using 45, 22.5 and 11.25 mg/kg) from which the lowest dose exhibiting the highest reproductive damages with no mortality was chosen (results not included). Doses of clomiphene citrate and vitamin E which are used against male infertility for their androgenic and antioxidant properties respectively were selected from a previous work [18, 19]. Doses of *L. acida* were chosen from our pilot study from which the calculated corresponding rat therapeutic dose was 340 mg/kg (unpublished); we also chose an infra therapeutic dose (170 mg/kg) for comparative approach.

### Sacrifice and sample collection

Twenty-four hours after each last treatment (day 15 or day 29), the corresponding rats were sacrificed (5/group) under diazepam/ketamine anesthesia. Blood was collected through abdominal artery and centrifuged for 15 min at 3000 rpm. The plasma was thereafter gently pipetted and kept in sealed tubes at − 20 °C prior to the measurement of sexual hormones. Reproductive organs were also collected for weight and spermatozoa parameter measurements as well as histological assessment.

### Measurement of the reproductive organ weights

After sacrifice, testis, prostate, seminal vesicles, vas deferens and epididymis were excised, freed from surrounding connective tissues and weighed.

### Epididymal spermatozoa motility and density

Immediately after sacrifice, the right cauda epididymis of each rat was minced in 10 ml of saline solution (0.9%) at 34 °C. To estimate the motility, 10 μl of a diluted solution were inserted in A (Ratio: 1/1) and B (Ratio: 1/2) chambers of the Mallassez hemocytometer and, motile and non-motile spermatozoa were counted in 10 random squares (5 in each chamber) using a light microscope (OLYMPUS, 40X). The result of each rat was expressed as the percentage of motile spermatozoa over the total number of spermatozoa [20].

For spermatozoa density, the solution obtained from the dilaceration of the cauda epididymis was kept at room temperature for 24 h to allow migration of spermatozoa from the tissue to the physiological solution. The solution was then diluted (1/11 ratio) and 10 μl were placed in A and B counting chambers of the Mallassez hemocytometer. The density was expressed as the total number of spermatozoa counted in 20 random squares using light microscope (OLYMPUS, 40X) [20].

### Spermatozoa viability and morphology

Spermatozoa smear was prepared using a drop of the solution obtained from the cauda epididymis and eosin-necrosin for staining. The preparation was dried at room temperature and a minimum of 200 spermatozoa were examined in 20 randomly selected fields using light microscope. Spermatozoa with stained cytoplasm were considered nonviable and the viability was calculated as the percentage of viable spermatozoa cells over the total number. For morphological abnormalities, spermatozoa cells with morphological abnormalities were counted and expressed as percentage of head and tail abnormalities as well as cytoplasmic droplets over the total number of cells [20].

### Sexual hormone measurements

Plasmatic testosterone, LH and FSH concentrations were measured by ELISA method according to the instructions of commercial kits (Accubind, Monobind. Lake Forest, USA).

### Assessment of oxidative stress markers

Testis homogenate was prepared at 10% in ice-cold 10 mM Tris buffer (pH 7.4) and centrifuged for 10 min at 3000 rpm at 4 °C. The supernatant was collected separately and stored at − 20 °C for biochemical analysis. Total proteins were quantified using bovine serum albumin as standard and lipid peroxidation (LPO) was measured in terms of malondialdehyde (MDA) production following the thiobarbituric acid method [21]. Superoxide dismutase (SOD) and Catalase (CAT) activities were measured as per described by Dimo et al. [22]. Total peroxidase activities were measured using the potassium iodate method [23].

### Testis histopathological evaluation

Immediately after sacrifice, rat left testis were fixed in 10% formaldehyde. Histological evaluation was performed following the method described by Tamizhazhagan and Pugazhendy [24]. Fixed material was washed out for 3-5 min in running tap water to remove excessive fixative solution. Pieces of testes were then passed through alcohol series for dehydration procedure and tissues were embedded in paraffin and cut into 5 μm thick sections using a rotary microtome. Slides were Hematoxylin-Eosin-stained before examination of the structure and diameter of seminiferous tubules using a light microscope (OLYMPUS, 400X).

### Statistical analysis

Results are presented as mean ± S.E.M. Two-way analysis of variance (ANOVA) and Tukey-HSD *post-hoc* test were used to determine statistical differences. Values were considered statistically significant if *P <* 0.05. STATISTICA/PC program (Version 8.0.) was used for data analysis.

## Results

### Effects of treatments on body weight

Contrary to the untreated group, a 14.42% decrease in body weight variation was observed in colibri®-exposed rats (Table 1). After 14 or 28 days of treatment with different pharmacological substances, a time-dependent recovery was noticed. Plant-treated rats recorded a remarkable body weight gain with the highest value (42.40%) observed in rats administered with the methanol extract of *L. acida* at 170 mg/kg for 28 days (Table 1).

**Table 1:** Effects of treatments on body weight gain in colibri®-exposed rats

Treatments	Induction period			Treatment period		
	Initial weight (g)	Final weight (g)	Variation (%)	Initial weight (g)	Final weight (g)	Variation (%)
14 Days						
Untreated	150.20 ± 1.85	167.80 ± 1.85	11.80 ± 2.09	167.80 ± 1.85	184.40 ± 3.70	9.89 ± 1.80
Colibri® + DW	175.20 ± 6.28	168.40 ± 5.99	−3.71 ± 2.92	168.40 ± 5.99	172.80 ± 8.28	2.61 ± 1.20
Colibri® + CC	174.80 ± 2.87	168.00 ± 3.08	−3.78 ± 2.40	168.00 ± 3.08	182.40 ± 3.66	8.56 ± 0.70
Colibri® + Vit E	175.20 ± 4.39	156.20 ± 3.80	−10.71 ± 2.37	156.20 ± 3.80	161.20 ± 5.89	3.21 ± 2.86
Colibri® + AE	178.40 ± 3.75	165.80 ± 5.84	−7.11 ± 2.20	165.80 ± 5.84	184.40 ± 6.31	11.51 ± 3.77
Colibri® + AE	184.60 ± 3.70	167.60 ± 8.45	−9.12 ± 4.56	167.60 ± 8.45	181.80 ± 8.39	8.66 ± 2.22
Colibri® + ME	177.60 ± 5.00	166.40 ± 4.34	−5.99 ± 3.87	166.40 ± 4.34	181.20 ± 5.99	9.20 ± 4.81
Colibri® + ME	175.40 ± 3.79	145.80 ± 6.04	−16.95 ± 2.47	145.80 ± 6.04	170.40 ± 8.02	17.14 ± 4.42
28 Days						
Untreated	153.40 ± 1.21	163.20 ± 3.12	6.38 ± 1.75	167.60 ± 6.82	179.00 ± 9.86	8.22 ± 2.16
Colibri® + DW	173.80 ± 4.15	151.40 ± 7.51	−12.98 ± 3.28	151.40 ± 7.51	177.00 ± 6.51	17.71 ± 5.90
Colibri® + CC	172.40 ± 1.57	147.20 ± 9.74	−14.66 ± 5.44	147.20 ± 9.74	181.00 ± 10.20	23.97 ± 6.98
Colibri® + Vit E	166.20 ± 1.74	149.20 ± 5.91	−10.27 ± 3.17	149.20 ± 5.91	180.60 ± 7.34	22.09 ± 7.97
Colibri® + AE	168.60 ± 1.75	133.60 ± 5.72	−20.84 ± 2.80	133.60 ± 5.72	191.20 ± 8.84	43.18 ± 2.92
Colibri® + AE	181.60 ± 5.90	139.00 ± 5.55	−23.30 ± 3.20	139.00 ± 5.55	192.40 ± 3.56	39.62 ± 7.90
Colibri® + ME	178.80 ± 4.13	133.80 ± 4.85	−25.15 ± 2.21	133.80 ± 4.85	190.20 ± 5.14	42.40 ± 2.33
Colibri® + ME	174.40 ± 3.44	136.20 ± 3.75	−21.81 ± 2.49	136.20 ± 3.75	186.80 ± 3.81	37.44 ± 3.59

Number of rats per group = 5. All values are expressed as mean ± SEM

*DW* distilled water, *CC* clomiphene citrate, *Vit E* vitamin E, *AE* aqueous extract, *ME* Methanol extract

### Effects of treatments on reproductive organ weights

#### Effects on testis and epididymis weights

A significant decrease in both testis (*p* ≤ 0.001) and epididymis (*p* ≤ 0.01) relative weights was recorded in the colibri®-exposed rats at all time points with reference to unexposed animals. On the contrary, all doses of the plant extracts significantly increased the relative weight of testis (*p* ≤ 0.001) and epididymis (*p* ≤ 0.05–0.001) when compared with colibri® + DW group (Table 2). Similar results were obtained with clomiphene citrate and vitamin E. Importantly, treatment with *L. acida* was more effective after 28 days and the most active dose was recorded with the aqueous extract at 170 mg/kg.

**Table 2:** Effects of *L. acida* on relative weight of reproductive organs in colibri®-exposed rats

Treatments	Doses	Relative weights (mg/100 g bw)			
		Testis	Epididymis	Seminal vesicle	Prostate
Untreated	10 ml/kg	1317.79 ± 55.90	385.47 ± 18.52	504.34 ± 44.17	124.73 ± 20.46
14 Days					
Colibri® + DW	10 ml/kg	1006.94 ± 57.68^###^	260.42 ± 28.01^##^	318.29 ± 28.57^##^	92.59 ± 6.01^#^
Colibri® + CC	2 mg/kg	1211.62 ± 40.68**	383.77 ± 15.52**	515.35 ± 41.57**	109.65 ± 3.83
Colibri® + Vit E	75 mg/kg	1439.21 ± 47.40***	372.21 ± 5.95**	409.43 ± 50.29	124.07 ± 11.98*
Colibri® + AE	170 mg/kg	1361.17 ± 31.18***	390.46 ± 15.10***	536.88 ± 32.46**	108.46 ± 8.54
Colibri® + AE	340 mg/kg	1446.64 ± 38.06***	379.54 ± 25.95**	511.55 ± 39.47**	126.51 ± 10.28*
Colibri® + ME	170 mg/kg	1396.25 ± 31.49***	369.76 ± 8.85**	485.65 ± 26.33*	121.41 ± 5.22*
Colibri® + ME	340 mg/kg	1519.95 ± 38.42***	363.85 ± 16.60*	498.83 ± 49.44*	105.63 ± 12.00
28 Days					
Colibri® + DW	10 ml/kg	1000.00 ± 59.98^###^	248.59 ± 26.46^##^	469.27 ± 45.33	122.91 ± 17.37
Colibri® + CC	2 mg/kg	1574.59 ± 24.17***	403.31 ± 13.84*	316.38 ± 23.12^#^	79.10 ± 8.31^#^
Colibri® + Vit E	75 mg/kg	1478.41 ± 50.77***	404.21 ± 17.89*	370.17 ± 33.46	77.35 ± 11.55
Colibri® + AE	170 mg/kg	1626.57 ± 48.17***	402.72 ± 76.59*	531.56 ± 71.37**	132.89 ± 17.14*
Colibri® + AE	340 mg/kg	1559.25 ± 61.10***	405.41 ± 29.78*	523.01 ± 74.79**	125.52 ± 22.76*
Colibri® + ME	170 mg/kg	1719.24 ± 23.00***	452.16 ± 14.78**	540.54 ± 48.29**	145.53 ± 16.37**
Colibri® + ME	340 mg/kg	1595.29 ± 32.51***	418.00 ± 10.04*	662.46 ± 43.99***	136.70 ± 7.49*

Number of rats per group = 5. All values are expressed as mean ± SEM

*DW* distilled water, *CC* clomiphene citrate, *Vit E* vitamin E, *AE* aqueous extract, *ME* Methanol extract

##: *p* ≤ 0.01; ###: *p* ≤ 0.001: significantly different compared with untreated animals; *: *p* ≤ 0.05; **: *p* ≤ 0.01: significantly different compared with colibri® + DW group; ***: *p* ≤ 0.001: significantly different compared with colibri® + DW group

#### Effects on seminal vesicles and prostate weights

Colibri® exposition decreased the relative weights of seminal vesicles (36.89 and 6.95%) and prostate (25.77 and 1.46%) glands after 14 and 28 days of treatment compared to untreated rats. Clomiphene citrate, vitamin E as well as the plant extracts remarkably reversed these drops as shown in Table 2. For instance, the aqueous and methanol extracts of *L. acida* (170 mg/kg) induced a significant increase (*p* ≤ 0.05–0.001) in prostate and seminal vesicles weights after 28 days of treatment.

### Effect of treatments on spermatozoa parameters

#### Effects on spermatozoa density and motility

**Fig. 1:**
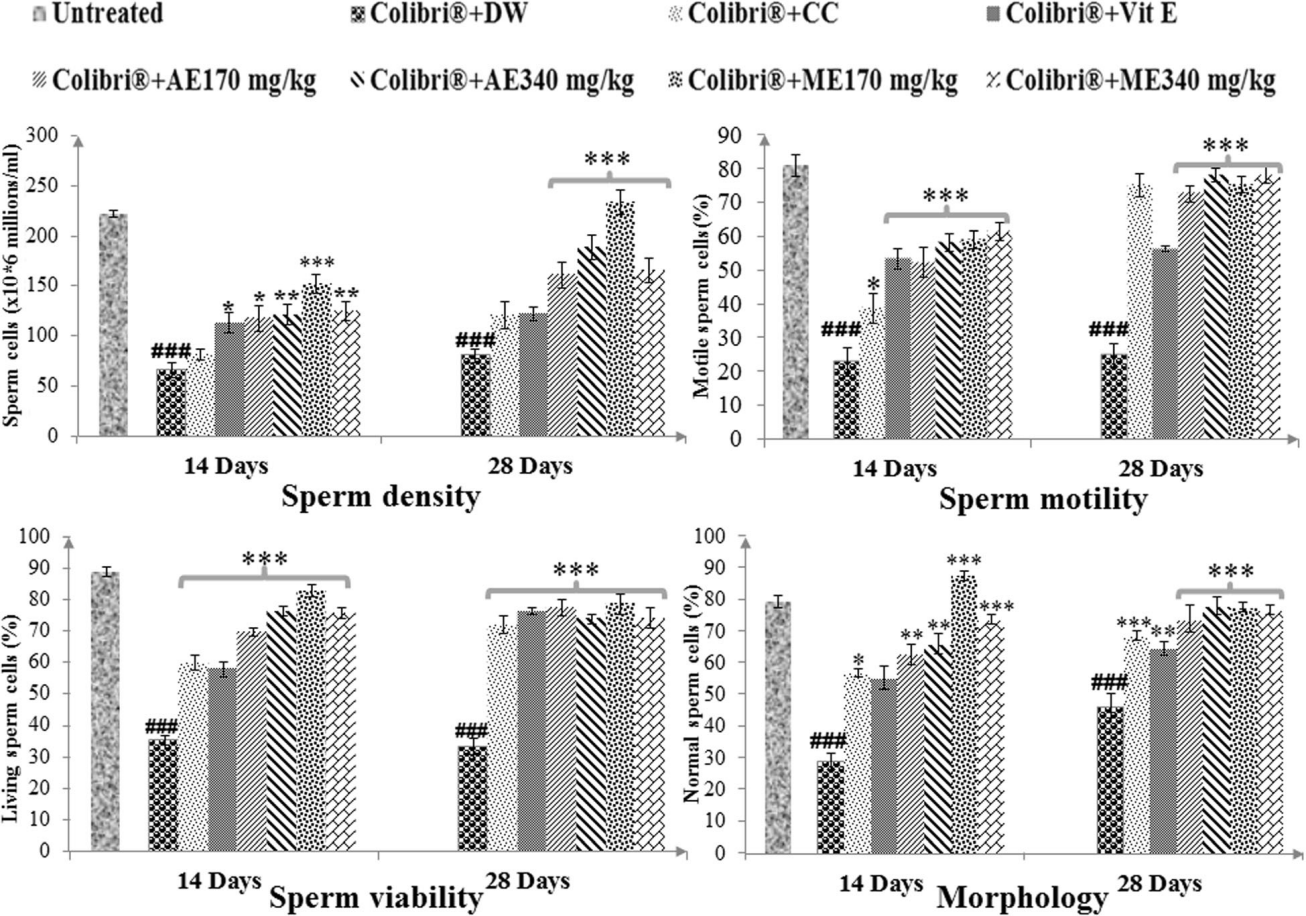
Effects of *L. acida* on spermatozoa density, motility, viability and morphology in colibri®-exposed rats. Number of rats per group = 5. All values are expressed as mean ± SEM. DW: distilled water; CC: clomiphene citrate; Vit E: vitamin E; AE: aqueous extract; ME: Methanol extract; ###: *p* ≤ 0.001 significantly different compared with untreated animals; *: *p* ≤ 0.05; **: *p* ≤ 0.01; ***: *p* ≤ 0.001 significantly different compared with colibri® + DW group

Colibri®-exposed rats showed a significant decrease in spermatozoa density (*p* ≤ 0.001) and motility (*p* ≤ 0.001) after 14 days compared with untreated rats. The aqueous and methanol extracts of *L. acida* significantly restored the epididymal spermatozoa density (*p* ≤ 0.05–*p* ≤ 0.001) and motility (*p* ≤ 0.001) in colibri® pre-exposed animals (Fig. 1). The doses 340 mg/kg of the aqueous and 170 mg/kg of the methanol extract showed highest activities after 28 days of continuous treatment.

#### Spermatozoa viability and morphology

The harmful effect of colibri® on male reproduction was further characterized by a significant decrease in spermatozoa viability associated to an increase (*p* ≤ 0.001) in spermatozoa morphological abnormalities including the following: head, tail abnormalities, tailless spermatozoa and cytoplasmic droplets (Fig. 1, Table 3). *L. acida* extracts significantly reversed these features concerning spermatozoa viability (increase, *p* ≤ 0.001) and spermatozoa morphology (increase, *p* ≤ 0.001) (Fig. 1). Also, a significant reduction (*p* ≤ 0.05–0.001) in the above-mentioned spermatozoa abnormalities was recorded in plant extracts-treated rats when compared with colibri® + DW group (Table 3).

**Table 3:** Effects of *L. acida* on spermatozoa abnormalities in colibri®-exposed rats

Treatments	Doses	Head abnormality	Tailless SPZ	Tail abnormality	Cytoplasmic droplet
Untreated	10 ml/kg	7.81 ± 0.25	0.60 ± 0.43	15.80 ± 0.92	0.34 ± 0.21
14 Days					
Colibri® + DW	10 ml/kg	32.00 ± 3.16^###^	5.99 ± 1.65	52.90 ± 1.16^###^	5.68 ± 1.81
Colibri® + CC	2 mg/kg	13.14 ± 1.04***	2.27 ± 0.48	34.52 ± 1.53***	2.68 ± 0.98
Colibri® + Vit E	75 mg/kg	27.84 ± 4.27	3.27 ± 0.78	23.19 ± 1.27***	4.36 ± 2.27
Colibri® + AE	170 mg/kg	11.30 ± 2.05***	3.54 ± 1.05	28.84 ± 2.51***	2.35 ± 0.64
Colibri® + AE	340 mg/kg	8.46 ± 1.01***	1.45 ± 0.46*	27.17 ± 1.69***	1.81 ± 0.61
Colibri® + ME	170 mg/kg	4.58 ± 1.28***	1.74 ± 0.31*	23.72 ± 1.16***	0.66 ± 0.31
Colibri® + ME	340 mg/kg	8.82 ± 1.50***	1.17 ± 0.33**	24.52 ± 1.56***	0.67 ± 0.17
28 Days					
Colibri® + DW	10 ml/kg	23.94 ± 2.54^###^	11.51 ± 6.40	40.55 ± 9.38^##^	3.96 ± 1.17^###^
Colibri® + CC	2 mg/kg	12.27 ± 2.53*	4.48 ± 1.97	20.87 ± 2.01***	0.69 ± 0.31**
Colibri® + Vit E	75 mg/kg	26.90 ± 4.13	2.23 ± 1.21	16.43 ± 1.45***	2.16 ± 0.38
Colibri® + AE	170 mg/kg	7.76 ± 1.30***	1.72 ± 0.77	14.71 ± 2.00***	0.67 ± 0.31**
Colibri® + AE	340 mg/kg	4.78 ± 0.50***	0.99 ± 0.30	17.08 ± 2.27***	1.30 ± 0.42*
Colibri® + ME	170 mg/kg	10.38 ± 0.83**	2.26 ± 1.35	15.12 ± 1.57***	0.34 ± 0.34***
Colibri® + ME	340 mg/kg	5.94 ± 1.41***	3.38 ± 0.68	15.21 ± 1.32***	0.79 ± 0.44**

Number of rats per group = 5. All values are expressed as mean ± SEM

*SPZ* spermatozoa, *DW* distilled water, *CC* clomiphene citrate, *Vit E* vitamin E, *AE* aqueous extract, *ME* Methanol extract

##: *p* ≤ 0.01; ###: *p* ≤ 0.001 significantly different compared with untreated animals *: *p* ≤ 0.05; **: *p* ≤ 0.01; ***: *p* ≤ 0.001: significantly different compared with colibri® + DW group

### Effect of different treatments on sexual hormones

**Fig. 2:**
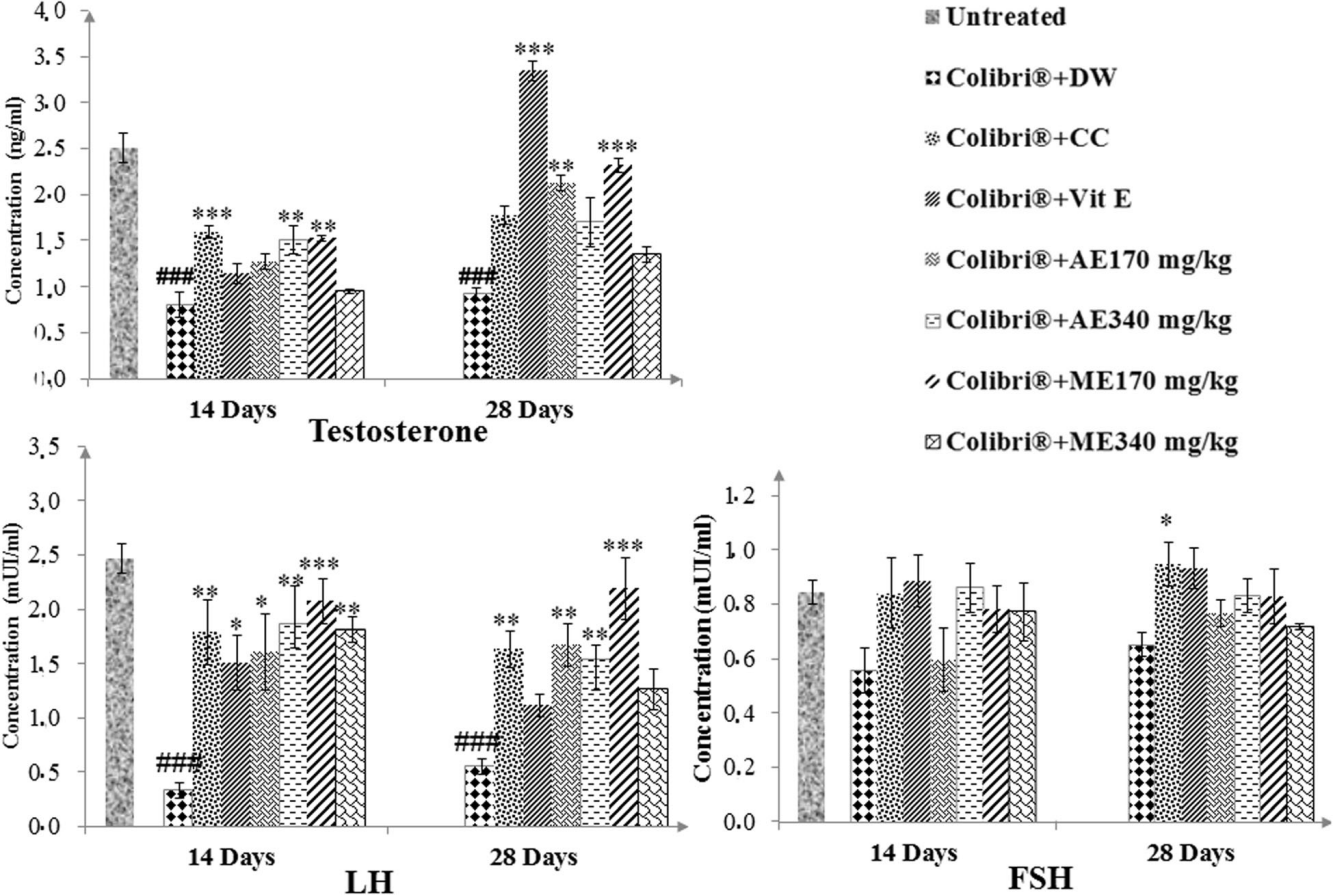
Effects of *L. acida* on reproductive hormones in colibri®-exposed rats Number of rats per group = 5. All values are expressed as mean ± SEM. DW: distilled water; CC: clomiphene citrate; Vit E: vitamin E; AE: aqueous extract; ME: Methanol extract; LH: Luteinizing hormone; FSH: Follicle Stimulating Hormone; ###: *p* ≤ 0.001 significantly different compared with control; *: *p* ≤ 0.05; **: *p* ≤ 0.01; ***: *p* ≤ 0.001: significantly different compared with Colibri® + DW group

The effects of various treatments on plasma testosterone, LH and FSH are indicated in Fig. 2. There was a significant decrease (*P* ≤ 0.001) in testosterone and LH levels in animals receiving Colibri®, FSH concentration was also lowered in negative control group (− 33.89% after 14 days) though no significant difference was noticed with regard to the untreated group. In Colibri®-pretreated rats, the aqueous and methanol extracts of *L. acida* significantly (*p* ≤ 0.05–0.001) restored the plasmatic contents of testosterone, LH and FSH. At all-time points, the methanol extract at 170 mg/kg showed the highest alleviating effect.

### Effects of different treatments on oxidative stress markers

#### Effects on total protein contents and lipid peroxidation

**Fig. 3:**
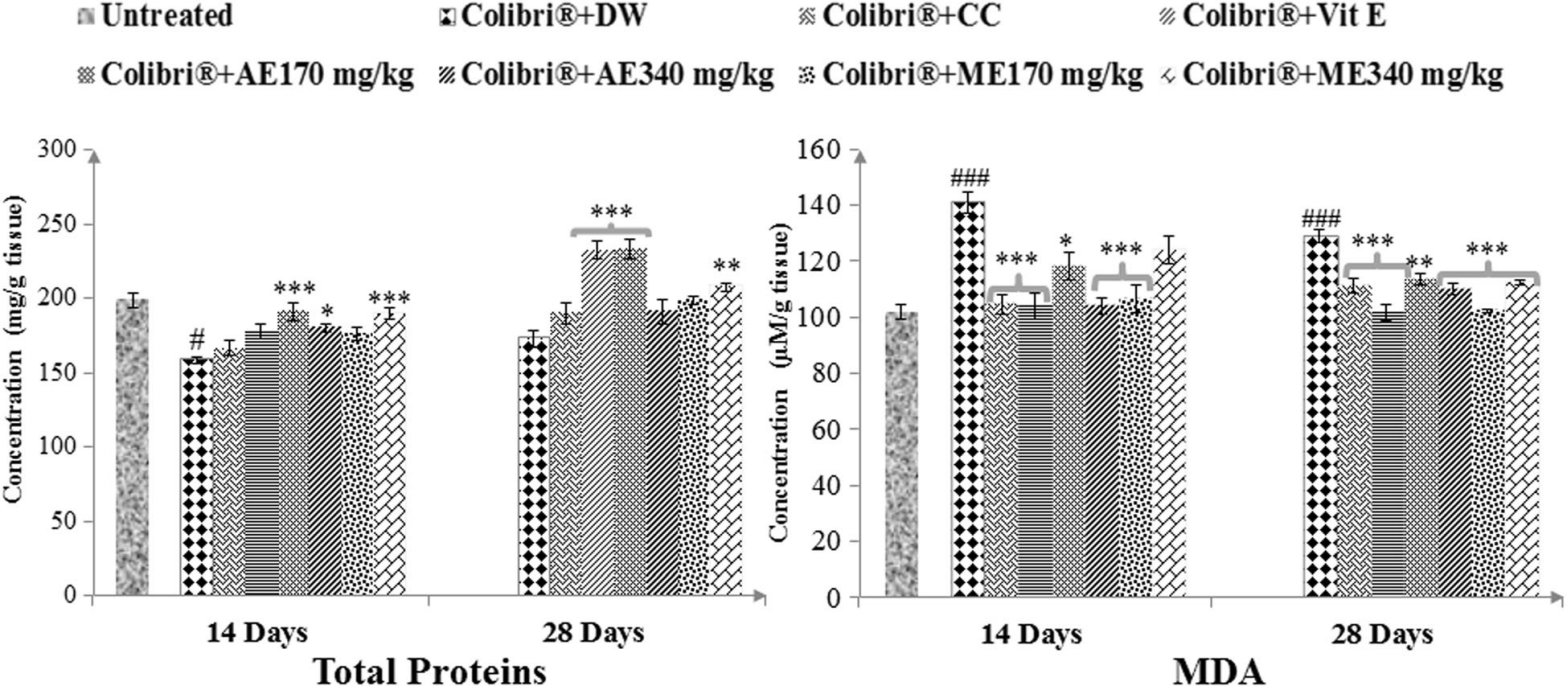
Effects of *L. acida* on testis total protein and lipid peroxidation in colibri®-exposed rats. Number of rats per group = 5. All values are expressed as mean ± SEM. DW: distilled water; CC: clomiphene citrate; Vit E: vitamin E; AE: aqueous extract; ME: Methanol extract; MDA: malondialdehyde; #: *p* ≤ 0.05; ###: *p* ≤ 0.001 significantly different compared with control; *: *p* ≤ 0.05; **: *p* ≤ 0.01; ***: *p* ≤ 0.001: significantly different compared with Colibri® + DW group

As expected, colibri® provoked a drop in testis total proteins and in lipid peroxidation (*p* ≤ 0.001) (Fig. 3). Aqueous and methanol extracts of *L. acida* as well as vitamin E brought out significant (*p* ≤ 0.05–0.001) changes through an increase in total proteins and a decrease in lipid peroxidation. Treatment with plant extracts was more effective after 28 days especially for the dose of 340 mg/kg for the aqueous and that of 170 mg/kg for the methanol extracts concerning total proteins and lipid peroxidation respectively.

#### Effects on SOD, CAT and total peroxidase activities

**Fig. 4:**
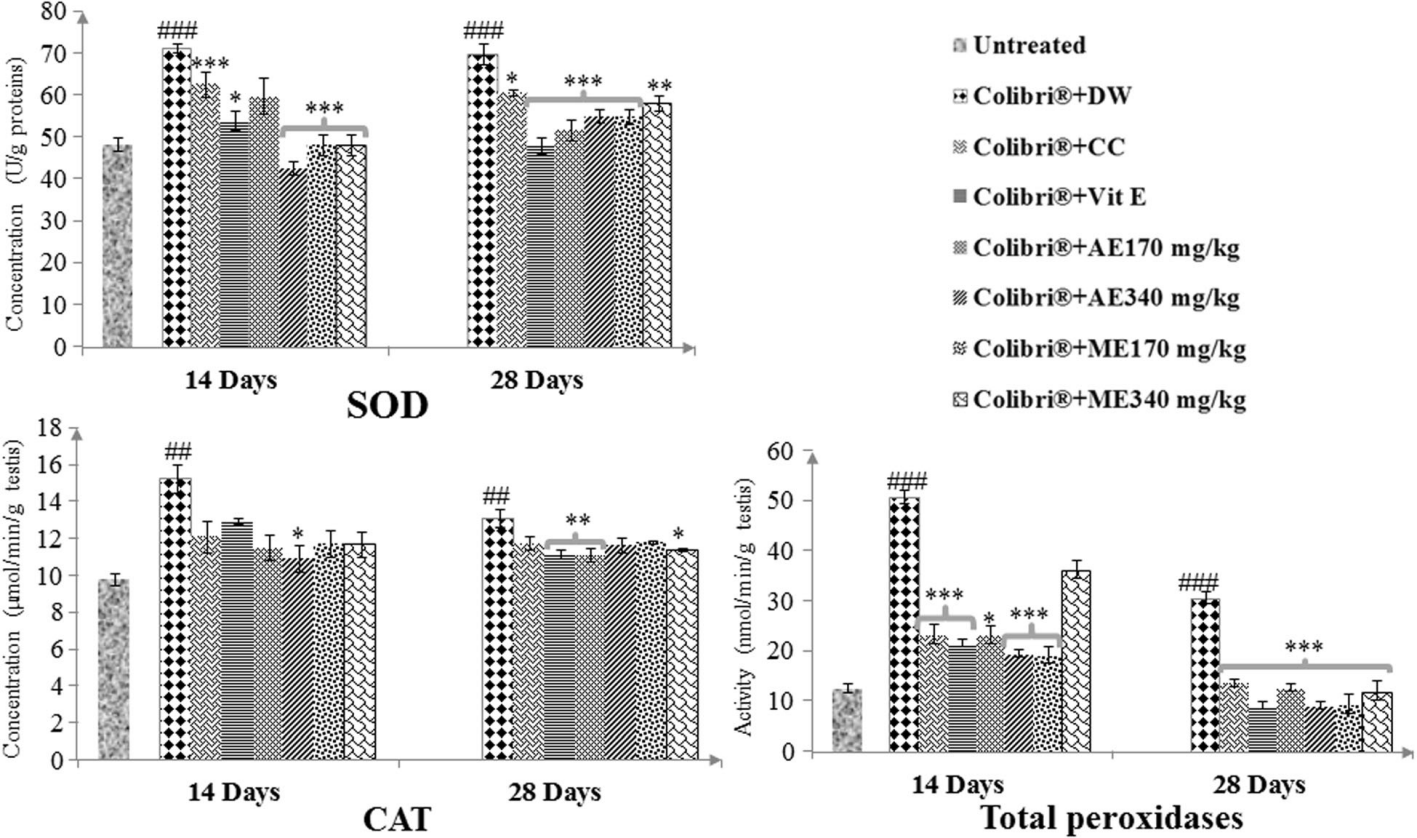
Effects of treatment SOD, CAT and total peroxidases activities in colibri®-exposed rats. Number of rats per group = 5. All values are expressed as mean ± SEM. DW: distilled water; CC: clomiphene citrate; Vit E: vitamin E; AE: aqueous extract; ME: Methanol extract; SOD: superoxide dismutase; CAT: catalase; ###: *p* ≤ 0.001 significantly different compared with control; *: *p* ≤ 0.05; **: *p* ≤ 0.01; ***: *p* ≤ 0.001: significantly different compared with colibri® + DW group

As shown in Fig. 4, colibri® exposition resulted in a significant increase (*p* ≤ 0.001) in SOD, CAT and total peroxidases (46.89, 55.66 and 310.48% respectively after 14 days) compared to the untreated group. On the contrary, after vitamin E and plant extract applications, a significant decrease (*p* ≤ 0.05–0.001) was noticed in the activity of SOD and total peroxidases. The aqueous extract at 340 mg/kg was the most active.

### Effects of treatments on testis histology

**Fig. 5:**
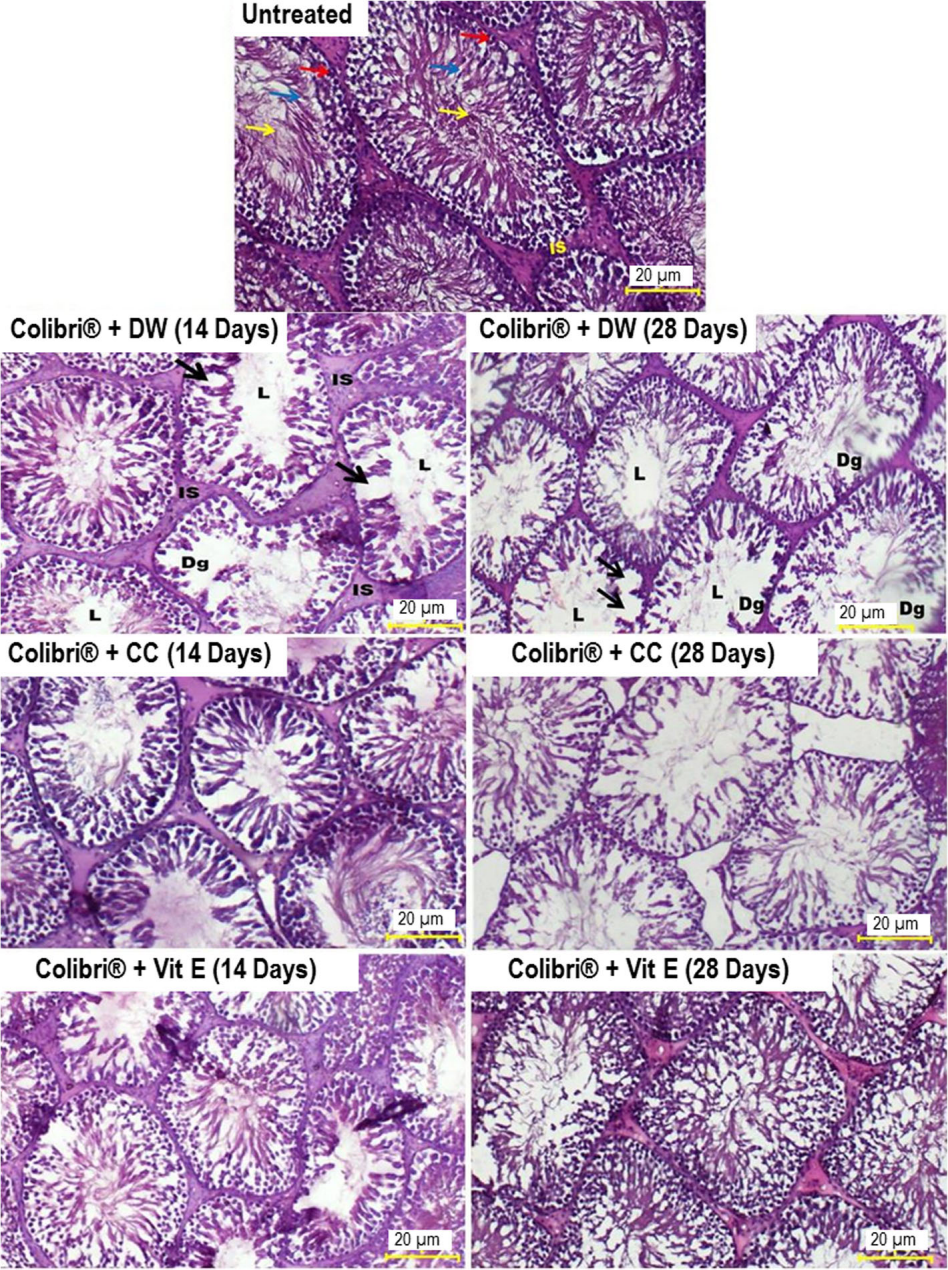
Effects of clomiphene citrate and vitamin E on testis histology in colibri®-exposed rats (*H&E X 400)*. Untreated rats and, clomiphene citrate and vitamin E-treated rats testes show complete spermatogenesis with spermatogonia (red arrow), spermatocyte (blue arrow) and spermatozoa (yellow arrow) and normal interstitial space (IS). Testes of colibri® + DW group show seminiferous tubules with wide interstitial space (IS), degeneration in the spermatogenic layer (Dg), vacuolation (black arrow) and few spermatozoa in lumen (L). DW: distilled water; CC: clomiphene citrate; Vit E: vitamin E

Testis analysis of untreated rats showed normal architecture consisting of well-organized seminiferous tubules, complete spermatogenesis and normal interstitial space (Fig. 5). However, testis sections of colibri® + distilled water rats revealed remarkable testicular damages marked by a significant reduction in seminiferous tubules diameter (*p* ≤ 0.01–*p* ≤ 0.001) (Table 4). Also, seminiferous tubules showed important degeneration, vacuolation and irregular basement membrane, incomplete spermatogenesis, increased interstitial space area with few spermatozoa in the lumen. Clomiphene citrate, vitamin E and *L. acida* extracts markedly reversed the above-mentioned histopathological changes particularly after 28 days of treatment (Table 4, Fig. 6).

**Table 4:** Effects of *L. acida* on the diameter of seminiferous tubules in colibri®-exposed rats

Treatment	Doses	Diameter of the seminiferous tubules (μm)	
		14 Days	28 Days
Untreated	10 ml/kg	402.82 ± 6.20	402.82 ± 6.20
Colibri® + DW	10 ml/kg	324.00 ± 7.6###	322.46 ± 7.92###
Colibri® + CC	2 mg/kg	349.26 ± 12.5	364.21 ± 3.92**
Colibri® + Vit E	75 mg/kg	354.93 ± 11.17	409.34 ± 7.17***
Colibri® + AE	170 mg/kg	338.27 ± 6.88	384.52 ± 9.20***
Colibri® + AE	340 mg/kg	372.40 ± 5.53**	389.14 ± 7.36***
Colibri® + ME	170 mg/kg	391.67 ± 7.65***	359.23 ± 8.62**
Colibri® + ME	340 mg/kg	324.34 ± 8.92	349.36 ± 7.35

Number of seminiferous tubules per group = 10. All values are expressed as mean ± SEM

*DW* distilled water, *CC* clomiphene citrate, *Vit E* vitamin E, *AE* aqueous extract, *ME* Methanol extract

###: *p* ≤ 0.001 significantly different compared with untreated animals; **: *p* ≤ 0.01; ***: *p* ≤ 0.001: significantly different compared with colibri® + DW group

**Fig. 6:**
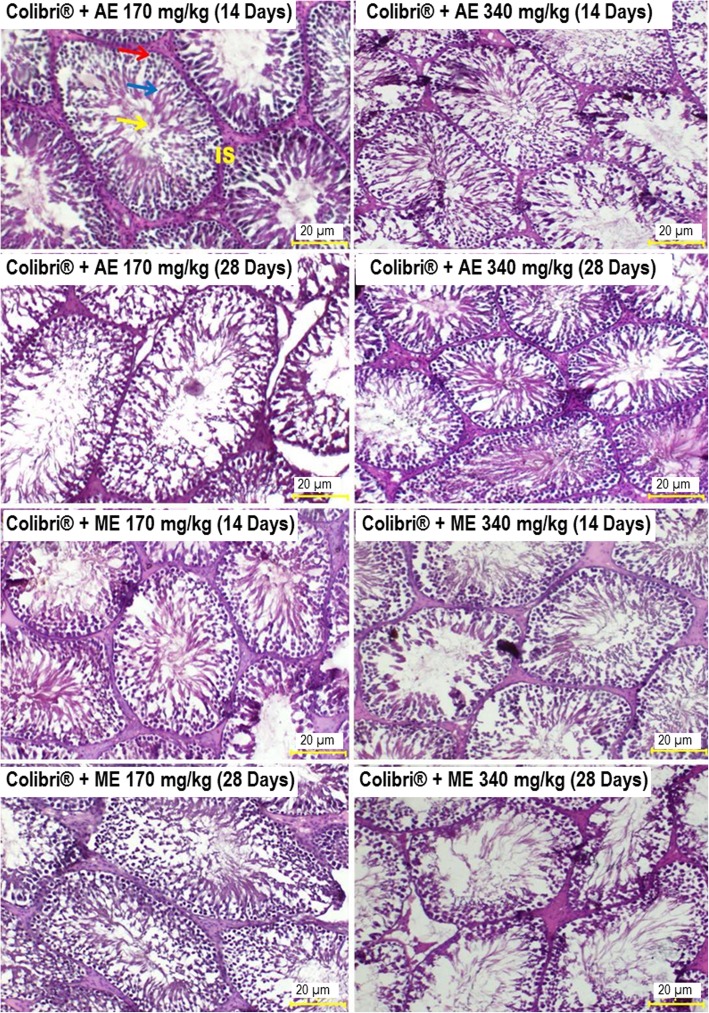
Effects of *L. acida* on testis histology in colibri®-exposed rats *(H&E X 400)*. *L. acida*-treated rats’ testes show complete spermatogenesis with spermatogonia (red arrow), spermatocyte (blue arrow) and spermatozoa (yellow arrow) and normal interstitial space (IS). AE: aqueous extract; ME: methanol extract

## Discussion

Pesticides toxicity against mammal systems is a serious matter of concern across continents. About 5% of the world’s population, mostly composed of agro-workers, is directly exposed to pesticides [4]. Toxic potentials of technical IMI, a neonicotinoid insecticide against mammal’s reproductive function, have been largely documented [5, 6, 25]. Some authors demonstrated that medicinal plants (broccoli) or plant-derived compounds (Ferulic acid or *Curcumin*) could be effective in preventing IMI toxicity [12, 13]. Contrary to previous reports which focused on the pure compound, the current study evaluated the reproductive toxicity of colibri®, a commercial formulation made of 3% IMI and the curative potentials of *L. acida,* an aphrodisiac medicinal plant, in adult male rats.

A significant decrease in body and sexual accessory organ weights was recorded in colibri®-exposed rats. The decrease in body weight gain could be due to the anorexic effects of IMI [26] while the drop in the testis, epididymis, seminal vesicles and prostate weights could be considered as a direct consequence of the decrease in testosterone and protein contents. It is well established that testosterone is the primary sex hormone with potent anabolic properties in various animal systems. In male, testosterone plays a key role in the development of male sexual organs and secondary sex characters including muscles and bone growth [27]. IMI, the active principle of colibri®, is a nicotinic receptor agonist which could also inhibit steroidogenesis. Kasson and Hsueh found that nicotine and its agonists exert an inhibitory effect on the 17-alpha hydroxylase, therefore preventing the conversion of pregnenolone and progesterone into precursors of DHEA and androstenedione respectively, thereby inhibiting testosterone synthesis in testis cells [28]. This detrimental effect of IMI on testosterone secretion has been previously reported [5, 25, 29].

Similar to clomiphene citrate, *L acida* significantly increased the body weight gain and the reproductive organs mass. Clomiphene citrate is a selective estrogen receptor modulator and is the common hormonal therapy for idiopathic male infertility [30]. By preventing the negative feedback imposed by estrogen on the hypothalamo-pituitary axis, this molecule increases the secretion of luteinizing hormone (LH) and subsequently testosterone synthesis [31]. The rise observed in testosterone concentration after plant extracts application could strongly justify these increases in body and organ weights. Present results corroborate the reported androgenic properties of the methanol extract of *L. acida* [15]. Flavonoids [16] found in *L. acida* extracts may partly justify this steroidogenic property [32–34].

Because spermatogenesis is closely dependent on steroidogenesis, and because the improvement of testis weight in plant-treated animals may be an indicator of normal spermatogenesis, we measured the spermatozoa parameters in the present study. A significant deterioration was noticed in spermatozoa quality and quantity of all colibri®-treated rats. Spermatogenesis is a physiological process regulated by both pituitary hormones and testosterone. Thus, LH coordinates the synthesis of testosterone which ensures growth and proliferation of germinal cells while FSH initiates the differentiation of these germinal cells into spermatogonia and spermatocytes. Together with FSH, testosterone controls the differentiation of spermatocytes into spermatids and spermatozoa [35]. Therefore, the deterioration of spermatozoa characteristics noted in this study could be attributed to the significant reduction in LH, FSH and testosterone. Our results are similar to those of Hahez et al. who demonstrated that in IMI-exposed rats, spermatozoa damage was associated with a decrease in sex hormones [9]. Moreover, this decrease in plasmatic LH and FSH could be partly explained by the oxidative potentials of IMI on the central nervous system [3].

Treatment of colibri®-exposed rats during 14 or 28 days with either clomiphene citrate, vitamin E or *L. acida* extracts significantly improved spermatozoa motility, density and viability. Moreover, plant-treated rats showed a significant decrease in spermatozoa abnormalities. Plant highest effects were recorded after 28 days of treatment with 340 mg/kg and 170 mg/kg of the aqueous and methanol extract respectively. These results may justify the increase observed in weights of the reproductive organs. As spermatogenesis relies on the reproductive hormones (FSH and testosterone) [35], the improvement of spermatozoa motility, density, viability and normal morphology observed in plant-treated rats corroborated with the rise of testosterone and FSH. Also, the significant decrease in spermatozoa morphological abnormalities could reflect a decrease in lipid peroxidation and oxidative stress, thereby suggesting the antioxidant properties of *L. acida* [16].

Oxidative stress is considered as the key mechanism behind the damaging effects of pesticides [36, 37]. The ingestion of IMI leads to the release of free radicals responsible of proteins and lipids peroxidation and, imbalance in the activities of endogenous antioxidant enzymes including SOD, CAT and peroxidases [38]. To investigate the involvement of the antioxidant properties of *L. acida* in the treatment of colibri®-related reproductive toxicity, some oxidative stress markers were quantified. Results showed that colibri® exposition resulted in a significant decrease in testis total proteins associated with a significant increase in lipid peroxidation and the activities of SOD, CAT and total peroxidases. These changes clearly support the damaging effects of IMI on testis tissues while the increase in the activities of the antioxidant enzymes indicates that colibri® exposition induced a moderate toxicity [39, 40]. This increase in oxidative stress parameters further justifies the above-mentioned severe spermatozoa damages. In fact, it has been shown that spermatozoa cells are particularly vulnerable to free radicals because of low concentrations of scavenging enzymes in their cytoplasm and the predominance of poly-unsaturated fatty acids in their membrane [41].

Treatment with clomiphene citrate, vitamin E and plant extracts remarkably regulated the aforementioned parameters. Vitamin E is a powerful antioxidant agent and is commonly required in male idiopathic infertility with increased amount of free radicals [18]. By normalizing the oxidant status, vitamin E protects testis cells from peroxidation and enhances steroidogenesis and spermatogenesis [42]. The decrease in oxidative stress markers in *L. acida*-treated rats, especially with the aqueous extract at 340 mg/kg, confirmed the antioxidant properties previously reported on this plant [16] in one hand, and corroborated the increase in sexual hormones and spermatozoa parameters in the other hand. Sobeh et al. showed that plants from *Lannea* genus contain important amount of catechins, a powerful scavenging agent to hydroxyl, peroxyl and superoxide radicals [43]. Moreover, a direct link between the amount of catechin and the antioxidant potential has been demonstrated [44]. Therefore, *L. acida* could contain similar compounds which count for the oxidative stress generated by colibri® metabolism, thereby protecting the hypothalamic-pituitary-testicular axis and promoting androgen synthesis.

In accordance with previous findings, light microscopic analyses of testis thin sections in this study showed a decreased diameter and severe damages in the seminiferous tubule’s architecture. These testis damages are a good indicator of the poor spermatozoa parameters observed in colibri®-exposed rats. Similar data were found by Soujanya et al. [8]. The normal testis histology was remarkably restored in animals treated during 28 days with plant extracts. As previously reported by other authors, histological damages recorded in the present study could be the result of tissue proteins and lipids peroxidation induced by IMI [5]. The alleviating effects of *L. acida* on testis histology could therefore be due to its antioxidant properties demonstrated in the present study [16].

Treatment of colibri®-exposed rats with aqueous or methanol extract of *L. acida* for 14 or 28 days significantly alleviated the harmful effects of colibri® on the male reproductive system. Plant extracts were generally more effective after 28 days of treatment with 340 mg/kg of the aqueous extract for oxidative stress parameters and 170 mg/kg of the methanol extract for sexual hormones and spermatozoa parameters.

## Conclusion

Results from the current study suggest that chronic exposure to colibri® induced severe testis toxicity. Aqueous and methanol extracts of *L. acida* markedly improved weight and spermatozoa parameters, sexual hormones and regulated antioxidant status in colibri®-exposed rats. These results are in line with the folk use of *L. acida* against infertility and suggest that this plant could be an effective alternative in the management of pesticides-derived reproductive toxicity.

## Data Availability

Data are available upon request.

## Abbreviations

CAT: Catalase
IMI: Imidacloprid
LPO: Lipid peroxidation
nAChRs: Nicotinic acetylcholine receptors
SOD: Superoxide dismutase
